# NMR resonance assignments of the EVH1 domain of neurofibromin’s recruitment factor Spred1

**DOI:** 10.1007/s12104-017-9768-1

**Published:** 2017-08-22

**Authors:** Sebastian Führer, Linda Ahammer, Angela Ausserbichler, Klaus Scheffzek, Theresia Dunzendorfer-Matt, Martin Tollinger

**Affiliations:** 1 Center for Molecular Biosciences Innsbruck (CMBI), Institute of Organic Chemistry, University of Innsbruck, Innrain 80/82, 6020 Innsbruck, Austria; 2 Division of Biological Chemistry, Biocenter, Medical University of Innsbruck, Innrain 80/82, 6020 Innsbruck, Austria

**Keywords:** NMR resonance assignment, TALOS+ prediction, Protein purification, Legius syndrome

## Abstract

Neurofibromin and Sprouty-related EVH1 domain-containing protein 1 (Spred1) both act as negative regulators of the mitogen-activated protein kinase pathway and are associated with the rare diseases Neurofibromatosis type 1 and Legius syndrome, respectively. Spred1 recruits the major GTPase activating protein (GAP) neurofibromin from the cytosol to the membrane in order to inactivate the small G protein Ras. These functions are dependent on the N-terminal EVH1 domain and the C-terminal Sprouty domain of Spred1 whereas the former specifically recognizes the GAP related domain of neurofibromin and the latter is responsible for membrane targeting. Within the GAP domain, Spred1 binding depends on the GAPex portion which is dispensable for Ras inactivation. In a first step towards the characterization of the Neurofibromin Spred1 interface in solution we assigned backbone and side chain ^1^H, ^13^C, and ^15^N chemical shifts of the Spred1 derived EVH1 domain. Our chemical shift data analysis indicate seven consecutive β-strands followed by a C-terminal α-helix which is in agreement with the previously reported crystal structure of Spred1(EVH1). Our data provide a framework for further analysis of the function of patient-derived mutations associated with rare diseases.

## Biological context

Spred1 (Sprouty-related EVH1 domain-containing protein 1) was originally identified as a negative regulator of the Ras driven mitogen-activated protein kinase (MAPK) pathway (Wakioka et al. 2001) which is dysregulated in numerous human malignancies. Spred proteins have been characterized as tumour suppressors in pediatric leukemia (Pasmant et al. 2015). Spred1 is also associated with the rare Legius syndrome (Brems et al. 2007, 2012) which is symptomatically related to Neurofibromatosis type1 (NF-1), a rare genetic disorder characterized by symptoms like the eponymous neurofibromas, skin pigmentation anomalies, learning disabilities, bone deformations, and an elevated risk to develop tumours of the peripheral and central nervous system (Riccardi 1992). NF-1 patients have mutations in the *NF1* gene encoding the 320 kDa major GTPase activating protein (RasGAP) (Ratner and Miller 2015; Upadhyaya and Cooper 2012). Tandem affinity purification experiments were performed in order to clarify the role of Spred1 in Ras inhibition and neurofibromin was identified as a component of a cellular complex including Spred1 (Stowe et al. 2012). Spred1 shares 55 and 38% sequence identity with Spred2 and Spred3, respectively, which all comprise an N-terminal Enabled/Vasodilator-stimulated phosphoprotein (VASP) homology 1 (EVH1) domain and a C-terminal sprouty-related (SPR) domain, whereas a central c-Kit binding domain is only present in Spred1 and Spred2 (Kato et al. 2003; Wakioka et al. 2001). The EVH1 domain was crucial for binding of cytosolic neurofibromin and the SPR domain was critical for its membrane recruitment. In our recent study (Dunzendorfer-Matt et al. 2016), we could identify the GAP related domain of neurofibromin as a direct binding site of the Spred1(EVH1) domain.

EVH1 domains are also present in other proteins, including members of the Enabled/VASP, the Homer/Vesl, and the Wiskott-Aldrich syndrome (WASP) families as well as the related Ran binding protein (RanBP) family which are implicated in signal transduction pathways, actin cytoskeletal re-organization, modulation of actin dynamics and actin-based agility. EVH1 domains have a pleckstrin homology like fold which is characterized by seven consecutive β-strands forming an antiparallel bent sheet along with a C-terminal α-helix (Scheffzek and Welti 2012) and have been described and structurally characterized as binding to proline-rich peptides with low affinity but high specificity (Ball et al. 2001; Barzik et al. 2001; Peterson and Volkman 2009; Prehoda et al. 1999; Renfranz and Beckerle 2002; Volkman et al. 2002). The structure of the frog and the human Spred1(EVH1) domains have been solved by X-ray crystallography [(Harmer et al. 2005); PDB: 3SYX, unpublished data].

Here we report the solution NMR backbone and side-chain assignment of the human Spred1(EVH1) domain. Our results are the first step towards the elucidation of the interface of a RasGAP with its membrane recruiting protein by NMR spectroscopy.

## Methods and experiments

### Sample preparation

Construction of pET-Spred1(EVH1) encoding the EVH1 domain of human Spred1 (Ser13–Ser130) preceded by a non-native translation initiating methionine and a glycine which was inserted due to the cloning strategy has been described (Dunzendorfer-Matt et al. 2016). The plasmid was transformed into the *E. coli* strain BL21(DE3) Star (Invitrogen). A starter culture (2.5 mL) was prepared in Luria Bertani (LB) medium containing 100 µg/mL carbenicillin, which was incubated at 37 °C and 220 rpm for 6–8 h, then diluted (1:20 (v/v)) into the same medium and kept overnight under the same conditions. The next day, cells were collected by centrifugation (2000×*g*) and resuspended in 1 L of minimal medium (M9) containing ^13^C_6_-D-glucose and/or ^15^NH_4_Cl (both Cambridge Isotope Laboratories), supplemented with 100 µg/mL carbenicillin. The culture was incubated at 37 °C and 220 rpm until the cell density reached about 0.2 measured photometrically at 600 nm. The temperature was shifted to 16 °C and incubation was continued overnight. Protein expression was induced by the addition of IPTG (isopropyl-β-D-1-thiogalactopyranosid, 1 mM) and performed for 3 h at 37 °C. Cells were harvested by centrifugation at 3200×*g* and 4 °C for 30 min, resuspended in 50 mM sodium phosphate buffer (pH 6.5) using 50 mL buffer per liter of bacterial culture multiplied with the absorption at 600 nm. Suspensions were shock-frozen in liquid nitrogen and stored at −80 °C until use. Expression as well as the following preparation steps of Spred1(EVH1) were monitored by SDS-PAGE. For lysate preparation, suspensions were pre-treated with Lysozyme and DNAse I (10 and 1 µg/mL final concentrations, respectively) and subsequently passed through a French press. The lysate was cleared by centrifugation (15,000×*g*, 4 °C, 40 min) and loaded onto a cation exchange column (HiTrap SP FF 5 mL, GE Healthcare). Elution of Spred1(EVH1) was achieved by applying a sodium chloride gradient in 50 mM sodium phosphate buffer (pH 6.5). Spred1(EVH1)-containing fractions were concentrated to a volume of 1–2 mL by centrifugation (Vivaspin Turbo 10 kDa MWCO, Sartorius) and loaded onto a size exclusion column (HiLoad 16/60 Superdex 75 prep grade, GE Healthcare) equilibrated in a buffer containing 20 mM sodium phosphate (pH 6.5), 50 mM sodium chloride, and 1 mM dithiothreitol. Spred1(EVH1)-containing fractions were pooled, concentrated to 0.6 or 0.8 mM (^15^N labeled or ^15^N/^13^C labeled samples, respectively) and supplemented with 10% D_2_O (v/v) for NMR data collection.

### NMR spectroscopy

All NMR experiments were performed at 25 °C on a 500 MHz Agilent DirectDrive 2 spectrometer equipped with a room temperature probe. For backbone resonance assignments ^1^H-^15^N-HSQC and three-dimensional HNCO, HNCACB and CBCA(CO)NH experiments were performed. Side-chain assignments were obtained by using ^1^H-^13^C-HSQC and three-dimensional (H)CC(CO)NH-TOCSY, H(CC)(CO)NH-TOCSY, ^1^H-^15^N-TOCSY-HSQC, ^1^H-^15^N-NOESY-HSQC and ^1^H-^13^C-NOESY-HSQC experiments. Collected data were processed with NMRPipe (Delaglio et al. 1995) and analyzed by using CcpNMR (Vranken et al. 2005) software.

### Assignments and data deposition

**Fig. 1:**
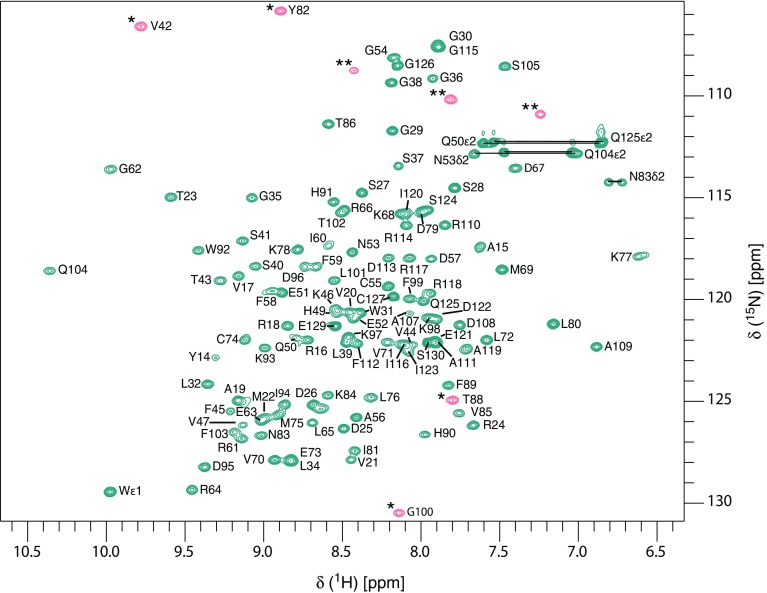
500 MHz ^1^H-^15^N-HSQC spectrum of Spred1(EVH1) (0.6 mM) in 20 mM sodium phosphate (pH 6.5), 50 mM sodium chloride, supplemented with 10% D_2_O. Data were recorded at 298 K. Assigned residues are indicated using *single letter codes*. Signals labeled by *asterisks* represent aliased signals, while those labeled with *two asterisks* likely derive from arginine side-chain ε-NH resonances. *Horizontal lines* indicate asparagine and glutamine side-chain NH_2_ resonances. *Numbering* of the Spred1(EVH1) domain corresponds to the full length human Spred1 sequence (NP_689807.1)

Excluding the N-terminal methionine and the non-native glycine we have assigned 112 of 113 non-proline residues in the ^1^H-^15^N-HSQC spectrum of Spred1(EVH1) (Fig. 1) corresponding to 99% completeness. Full (100%) assignment of C^α^ and C^β^ resonances was achieved, while backbone C’ assignments are 95% complete. In addition, 69, 62, and 30% of side-chain C^γ^, C^δ^, and C^ε^, respectively were assigned. Regarding protons, 99% of the H^α^ and H^β^ resonances along with 93, 67, and 50% of H^γ^, H^δ^, and H^ε^ resonances, respectively, were assigned. Spred1(EVH1) chemical shift data have been deposited at the Biological Magnetic Resonance Data Bank (http://www.bmrb.wisc.edu) under the Accession Number 27162.

**Fig. 2:**
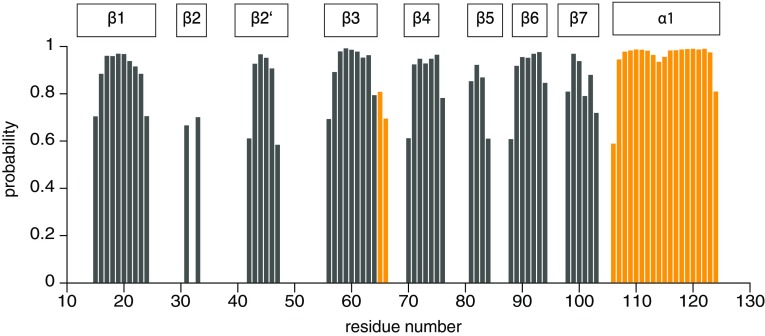
Probabilities for a secondary structure (*yellow*, α-helices; *grey*, β-strands) predicted from backbone chemical shifts (H^N^, N, C’, C^α^, and C^β^) are plotted as a function of residue numbers. Probability values below 0.4 (helices) or 0.35 (strands) are not displayed. Positions of secondary structure elements according to the crystal structure (PDB entry 3SYX) are indicated on *top*

A TALOS+ prediction (Shen et al. 2009) was conducted using the H^N^, N, C’, C^α^, and C^β^ chemical shifts of the protein and indicated secondary structure elements of Spred1(EVH1) (Fig. 2) that are consistent with the crystallographic data (Harmer et al. 2005; PDB: 3SYX, unpublished data), i.e. seven consecutive β-strands (β1–β7), where β2 is split into two segments β2 and β2′, along with the C-terminal α-helix. Of note, the NMR chemical shift data indicate that the loop between strands β3 and β4 has a moderate propensity for α-helical structure. In future experiments we will use the chemical shift data and address the function of patient-derived mutations in the interaction with neurofibromin.
